# Childhood maltreatment and the medical morbidity in bipolar disorder: a case–control study

**DOI:** 10.1186/s40345-017-0099-z

**Published:** 2017-09-07

**Authors:** Georgina M. Hosang, Helen L. Fisher, Rudolf Uher, Sarah Cohen-Woods, Barbara Maughan, Peter McGuffin, Anne E. Farmer

**Affiliations:** 1 Psychology Department, Goldsmiths, University of London, Lewisham Way, London, SE14 6NW UK; 2 MRC Social, Genetic and Developmental Psychiatry Centre, Institute of Psychiatry, Psychology & Neuroscience, King’s College London, De Crespigny Park, London, SE5 8AF UK; 3 Department of Psychiatry, Dalhousie University, 5909 Veterans’ Memorial Lane, Halifax, NS B3H 2E2 Canada; 4 School of Psychology, Flinders University, GPO Box 2100, Adelaide, SA 5001 Australia

**Keywords:** Bipolar disorder, Childhood maltreatment, Medical illness, Physical health, Child abuse, Child neglect, Childhood adversity

## Abstract

**Background:**

Childhood maltreatment (abuse and neglect) can have long-term deleterious consequences, including increased risk for medical and psychiatric illnesses, such as bipolar disorder in adulthood. Emerging evidence suggests that a history of childhood maltreatment is linked to the comorbidity between medical illnesses and mood disorders. However, existing studies on bipolar disorder have not yet explored the specific influence of child neglect and have not included comparisons with individuals without mood disorders (controls). This study aimed to extend the existing literature by examining the differential influence of child abuse and child neglect on medical morbidity in a sample of bipolar cases and controls.

**Methods:**

The study included 72 participants with bipolar disorder and 354 psychiatrically healthy controls (average age of both groups was 48 years), who completed the Childhood Trauma Questionnaire, and were interviewed regarding various medical disorders.

**Results:**

A history of any type of childhood maltreatment was significantly associated with a diagnosis of any medical illness (adjusted OR = 6.28, 95% confidence intervals 1.70–23.12, *p* = 0.006) and an increased number of medical illnesses (adjusted OR = 3.77, 95% confidence intervals 1.34–10.57, *p* = 0.012) among adults with bipolar disorder. Exposure to child abuse was more strongly associated with medical disorders than child neglect. No association between childhood maltreatment and medical morbidity was detected among controls.

**Conclusions:**

To summarise, individuals with bipolar disorder who reported experiencing maltreatment during childhood, especially abuse, were at increased risk of suffering from medical illnesses and warrant greater clinical attention.

## Background

Bipolar disorder is associated with substantial morbidity and mortality (Kupfer 2005); for instance people with bipolar disorder die up to 14 years younger than the general population (Chang et al. 2011). Premature mortality among individuals with this illness cannot be explained by suicide alone (Hoang et al. 2011) but has been attributed to the high rates of medical illnesses in this group (Crump et al. 2013). The rates of a myriad of medical disorders (e.g. diabetes, stroke, thyroid disease and cardiovascular disease) have been reported to be significantly elevated among people with bipolar disorder compared to the general population (Forty et al. 2014; Perron et al. 2009).

Little is known about the factors that are associated with the high medical burden in bipolar disorder, although the implications are huge for the individual, their families and society as a whole. The available evidence suggests that multiple factors are likely to contribute to the medical morbidity in bipolar disorder (and other serious mental illnesses), the factors which have received the most attention include side effects of psychotropic medications, unhealthy life style choices and issues with health care provision for this group. Various side effects are associated with psychotropic medication, such as mood stabilisers and antipsychotics which are commonly used to treat bipolar disorder, the most pertinent to this context include weight gain and insulin resistance (Newcomer 2007). Such side effects are risk factors for diabetes and cardiovascular disease (Correll et al. 2015) and thus may explain the high rates of these illnesses in people with bipolar disorder. Smoking, unhealthy diet and physical inactivity are lifestyle choices and habits which are prevalent in people with bipolar disorder (Scott and Happell 2011) but are also known risk factors for physical illnesses, such as diabetes and coronary heart disease, thus these lifestyle choices may explain the comorbidity between these illnesses and bipolar disorder (De Hert et al. 2011). A small but growing body of research suggests that people with serious mental illnesses are less likely to receive standard levels of care (De Hert et al. 2011). For instance, low rates of surgical interventions for coronary heart disease (e.g. stenting) and screening for metabolic abnormalities associated with diabetes are recorded for people with mental illnesses including bipolar disorder (De Hert et al. 2011). This is despite the fact that such illnesses are highly prevalent in this population. A factor which has received less attention in this context is the influence of childhood adversity. Preliminary research suggests that the experience of childhood maltreatment may contribute to the medical morbidity observed in bipolar disorder (Post et al. 2013), but these findings await replication and extension.

Childhood maltreatment encompasses both abuse (e.g. sexual, emotional and physical abuse) and neglect (lack of provision for the individual’s needs by their care-giver, including food, shelter and support) (Norman et al. 2012). Childhood maltreatment can be considered a plausible risk factor for the comorbidity between medical illnesses and bipolar disorder based on two lines of evidence. First, childhood maltreatment is associated with lasting changes or abnormalities in a number of biological systems or processes detected in adulthood (Gonzalez 2013; Danese and Lewis 2016). For instance, increased inflammatory cytokines are exhibited by maltreated individuals both as children (Slopen et al. 2013) and adults (Baumeister et al. 2016). Elevated inflammation has also been implicated in bipolar disorder (Leboyer et al. 2012) and a series of medical illnesses, such as diabetes, arthritis and certain cancers (Couzin-Frankel 2010) and thus could explain the comorbidity between the two disorder groups. Moreover, there is evidence that people with mood disorders and a history of childhood maltreatment exhibit particularly pronounced elevation in inflammation levels (Danese et al. 2008, 2011). For example, maltreated individuals with depression have significantly increased inflammation levels compared to those with depression only, a history of childhood maltreatment only and those without either (controls) (Danese et al. 2011).

Secondly, childhood maltreatment is associated with an increased risk of medical illnesses (Hosang et al. 2013; Scott et al. 2011) and bipolar disorder (Fisher and Hosang 2010; Palmier-Claus et al. 2016) in adulthood. The results from several studies have gone further and shown that childhood maltreatment is linked to the co-occurence of medical illnesses and mood disorders (including bipolar disorder) (Lu et al. 2008; McIntyre et al. 2012). To date only one study has examined this relationship in bipolar disorder specifically and found that childhood adversity is significantly related to the diagnosis of medical illnesses in adulthood, including diabetes, cardiovascular disease and asthma (Post et al. 2013).

The limited available research in this area has not explored the specific role of child neglect but has focused on broadly defined childhood maltreatment (McIntyre et al. 2012), or childhood adversity which includes child abuse, parental psychopathology and violence in the home (Lu et al. 2008; Post et al. 2013). Exposure to child neglect has been related to a number of medical illnesses, such as cardiovascular disease, diabetes and osteoarthritis in adulthood (Norman et al. 2012; Scott et al. 2011) and therefore is a crucial construct to consider in this context. Furthermore, previous studies examining the medical morbidity in bipolar disorder have not included comparisons with control groups (Post et al. 2013), thus it remains unclear whether the relationship between child adversity (including childhood maltreatment) and medical illnesses is specific to or greater among people with bipolar disorder relative to the general population.

To address the methodological gaps in the literature, the current study aimed to investigate the association between a history of child maltreatment and the diagnosis of medical illnesses in adulthood among people with bipolar disorder compared to unaffected controls (those without a personal or family history of a psychiatric illness). The differential influence of child abuse and child neglect on the diagnosis of medical illnesses was also examined in this context. It is hypothesised that both child abuse and neglect will be more significantly associated with medical illnesses in the bipolar disorder group compared to controls.

## Methods

### Participants

A total of 426 participants were included in this study, 354 (58% females, *N* = 205) of which were psychiatrically healthy controls and 72 (78% females, *N* = 56) were diagnosed with bipolar disorder (see Table 1). The participants with bipolar disorder were aged between 29 and 72 years, with a mean of 48.4 years (SD = 9.43). Participants with bipolar disorder were enrolled in the BADGE (gene-environment interplay in bipolar affective disorder) study (see Hosang et al. 2012) and were recruited by re-contacting bipolar cases from the Bipolar affective disorder case–control study (BaCCs) (see Gaysina et al. 2009; Hosang et al. 2017). Participant recruitment for BACCs was mainly via psychiatric outpatient clinics with the rest enlisted through media advertisement and self-help groups in the UK. All participants with bipolar disorder met DSM-IV criteria for bipolar I or bipolar II disorder ascertained via the schedules for clinical assessment in neuropsychiatry interview (see “Measures” section), and were Caucasian to control for population stratification since they were originally recruited from a genetic association study (see Gaysina et al. 2009). Participants were excluded if their bipolar episodes only occurred in relation to substance misuse or a physical disorder or if they had a personal or family history of schizophrenia. Participants with bipolar disorder were not experiencing a mood episode at either of their assessments for the BaCCs and BADGE studies.

**Table 1:** Comparison of reports of each medical condition and history of childhood maltreatment between bipolar disorder cases and unaffected controls

	Bipolar cases, *N* (%) [*N* = 72]	Controls, *N* (%) [*N* = 354]	Statistic	*p* value
Female	56 (78)	205 (58)	*χ* ^2^(1) = 9.34	*0.002*
Age at assessment [mean (SD)]	48.36 (9.42)	47.73 (9.15)	*t* (395) = 0.52	0.60
At least one medical illness	42 (58)	96 (27)	*χ* ^2^(1) = 26.61	*<0.001*
Number of medical illnesses				
None	30 (42)	259 (73)		
1	21 (29)	78 (22)		
2 or more	21 (29)	17 (4)	*χ* ^2^(2) = 49.88	*<0.001*
Different types of medical illnesses				
Heart problems (i.e. stroke, angina and heart attack)	1 (1)	2 (1)	Two-tailed fisher’s exact	0.427
Asthma	15 (21)	31 (9)	*χ* ^2^(2) = 9.06	*0.003*
Diabetes (types I and II)	5 (7)	7 (2)	Two-tailed fisher’s exact	*0.036*
Arthritis	17 (24)	23 (7)	*χ* ^2^(2) = 20.60	*<0.001*
Hypertension	16 (22)	32 (9)	*χ* ^2^(2) = 10.40	*0.001*
Epilepsy or convulsions	3 (4)	0 (0)	Two-tailed fisher’s exact	*0.005*
Osteoporosis	3 (4)	3 (0.9)	Two-tailed fisher’s exact	0.063
Multiple sclerosis	0 (0)	0 (0)	–	
Emphysema or chronic bronchitis	5 (7)	14 (4)	*χ* ^2^(2) = 1.26	0.263
Postherpetic neuralgia	0 (0)	2 (0.6)	Two-tailed fisher’s exact	1.00
Childhood maltreatment				
Any type of childhood maltreatment (abuse and/or neglect)^a, b^	37 (51)	64 (18)	*χ* ^2^(1) = 36.70	*<0.001*
Any type of child abuse^a, c^	31 (43)	41 (12)	*χ* ^2^(1) = 42.20	*<0.001*
Physical abuse	8 (11)	13 (4)	*χ* ^2^(1) = 6.99	*0.008*
Emotional abuse	20 (28)	23 (7)	*χ* ^2^(1) = 35.13	*<0.001*
Sexual abuse	20 (28)	20 (6)	*χ* ^2^(1) = 34.16	*<0.001*
Any type of child neglect^a, d^	20 (28)	36 (10)	*χ* ^2^(1) = 16.25	*<0.001*
Physical neglect	10 (14)	64 (6)	*χ* ^2^(1) = 5.04	*0.025*
Emotional neglect	19 (26)	28 (8)	*χ* ^2^(1) = 20.71	*<0.001*

Significant *p* values are italicised

*N* number of participants, *SD* standard deviations, % percentage, *P* probability due to chance

^a^These figures are not the sum of the derived variables as some participants report experiencing more than one type of maltreatment

^b^Childhood maltreatment was considered present if any type of child abuse or neglect were rated as moderate or severe

^c^Child abuse was considered present if any form of child abuse was rated as moderate or severe

^d^Child neglect was considered present if physical or emotional neglect was rated as moderate or severe

The controls were a sub-sample of a case–control genetic association study on unipolar depression that provided information on their experience of maltreatment during childhood (see Fisher et al. 2013). The controls were aged between 24 and 68 years with a mean of 47.7 years (SD = 9.15). They were recruited through UK general medical practices and excluded if they had a personal or family history (among first degree relatives) of any psychiatric disorder. Given that participants were drawn from genetic association studies they were Caucasian to control for population stratification. All participants were aged 18 years or over and provided written informed consent after the nature of the study and procedures were fully explained. All studies received ethical approval from either King’s College Hospital or the Joint from South London and Maudsley and Institute of Psychiatry Research Ethics Committees. All procedures contributing to this work were conducted in accordance with the Declaration of Helsinki in 1975 (revised in 2008), and the ethical standards of the national and institutional committees on human experimentation.

### Measures

#### Bipolar disorder diagnosis

The Schedules for Clinical Assessment in Neuropsychiatry (SCAN), Version 2.1 interview (Wing et al. 1990) was used to ascertain a lifetime DSM-IV diagnosis of bipolar disorder. The presence and severity of the psychopathology items were rated for the worst depressive and manic episodes, separately.

#### History of childhood maltreatment

All participants completed the 28-item Childhood Trauma Questionnaire (CTQ) (Bernstein et al. 2003), which was used to record the experience of five types of childhood maltreatment (i.e. physical abuse, sexual abuse, emotional abuse, physical neglect and emotional neglect). A total of 5 items were used to measure each type of maltreatment, which were rated on a 5-point Likert scale ranging from 1 (never true) to 5 (very often true). The cut-offs for moderate to severe levels of each type of maltreatment were employed in this study in accordance with the manual (Bernstein et al. 2003). The five types of childhood maltreatment rated as moderate or severe were categorised into abuse (i.e. sexual, emotional and/or physical abuse) and neglect (emotional and/or physical neglect). Good psychometric properties have been reported for this instrument, for instance there is high concordance between CTQ scores and therapists' ratings of childhood maltreatment (Bernstein et al. 2003). Moreover, good test–retest reliability has been found using this instrument in a sample of people with bipolar disorder (Shannon et al. 2016).

#### Medical history

All participants completed a self-report questionnaire to determine the lifetime presence of various medical illnesses (Farmer et al. 2008; Forty et al. 2014). Participants were asked whether they had been formally diagnosed with any of the following illnesses: heart problems (i.e. stroke, angina and heart attack), asthma, diabetes (I and II), arthritis (i.e. osteoarthritis, rheumatoid arthritis and other types of arthritis), hypertension, epilepsy or convulsions, osteoporosis, multiple sclerosis, emphysema or chronic bronchitis, or post herpetic neuralgia. Trained research assistants administered the questionnaire to all participants, which involved confirming that a formal diagnosis of the illness was provided by a medical professional (e.g. General Practitioner or medical consultant). Good concordance between the self-report of medical illnesses using this interview and practitioner ratings have been found (Farmer et al. 2008).

### Analyses

Group differences were tested using Chi-square (*χ* ^2^) tests, one-way ANOVAs and independent sample *t* tests. The Fisher’s exact test was conducted if a *χ* ^2^ test could not be used (e.g. expected values were less than 5). Case–control differences concerned with the association between childhood maltreatment and medical illnesses were examined using two approaches. First, using logistic regression models when at least one medical disorder was examined and second, ordinal logistic regression models when the number of medical illnesses was the focus (none, 1 and 2 or more illnesses). Gender and age were entered as covariates, along with child maltreatment, bipolar disorder status, as well as the interaction between childhood maltreatment and bipolar disorder status. Three parallel models (for each approach) were undertaken to investigate the effect of any type of childhood maltreatment, child abuse and child neglect. Given the relatively small bipolar disorder sample size and uneven distribution of some variables, we estimated the variance in the regression models with non-parametric bootstrap with replacement (1000 replications) to obtain empirical standard error estimates without making distributional assumptions. All statistical tests were performed in STATA version 14.0; the conventional level of significance, *p* < 0.05, was used in this study.

## Results

There was no significant age difference between the controls and participants with bipolar disorder (*t*(393) = 0.60, *p* = NS), but there was a significantly higher proportion of females in the bipolar group relative to the controls (*χ* ^2^(1) = 9.34, *p* = 0.002). The percentage of participants reporting each medical illness and different types of childhood maltreatment are presented in Table 1. The most commonly reported medical illnesses in the sample were arthritis, asthma and hypertension. The relatively low number of participants that recorded being diagnosed with each medical illness prevented the examination of associations between specific disorders and childhood maltreatment. Thus the remaining analyses focused on either the diagnosis of *at least one* or *the number of* (none, 1 and 2 or more) medical illnesses.

There were no gender differences in the diagnosis of at least one (*χ* ^2^(1) = 0.08, *p* = NS) or the number of medical disorders (*χ* ^2^(1) = 0.29, *p* = NS). Those individuals that reported receiving a diagnosis of at least one medical disorder were significantly older than those without a diagnosis (*t*(393) = 4.05, *p* < 0.001). A similar pattern was observed when the number of medical illnesses were examined (*F*(2, 392) = 9.92, *p* < 0.001): participants that reported 1 (mean age = 49.73 years, SD = 8.14) or at least 2 (mean age = 52.54 years, SD = 9.54) medical disorders were significantly older than the individuals that recorded none (mean age = 46.53 years, SD = 9.19) according to a Tukey post hoc test (*p* = 0.009, *p* < 0.001, respectively).

Significantly more participants with bipolar disorder reported being diagnosed with at least one medical illness compared to controls (*χ* ^2^(1) = 26.61, *p* < 0.001) and they also reported to have significantly more medical illnesses relative to controls (*χ* ^2^(2) = 49.88, *p* < 0.001). The rates of all types of childhood maltreatment were significantly greater among the bipolar group compared to the controls (see Table 1). A moderate correlation between child abuse and neglect was detected in the entire sample (Pearson’s *r*(426) = 0.33, *p* < 0.001).

Logistic regression models were conducted to explore the interaction between bipolar disorder status and the history of childhood maltreatment on the diagnosis of at least one medical illness, and ordinal logistic regression models were undertaken to examine the number of medical disorders (gender and age were included as covariates in the analyses), the results of which are presented in Table 2. Bipolar disorder status significantly interacted with both the exposure to any type of childhood maltreatment and child abuse on the diagnosis of at least one and the increased number of medical illnesses (see Table 2). The results remained significant for child abuse even when the effects of child neglect were controlled for (at least one medical illness: adjusted OR = 5.90, 95% confidence intervals (CI) 1.31–26.62, *p* = 0.021; number of medical disorders: adjusted OR = 4.85, 95% CI 1.30–18.06, *p* = 0.019). Although exposure to child neglect was associated with higher odds of having a medical illness with bipolar disorder, the results failed to reach conventional levels of significance (at least one medical illness: adjusted OR = 4.32, 95% CI 0.96–19.47, *p* = 0.057; number of medical disorders: adjusted OR = 3.30, 95% CI 0.89–12.22, *p* = 0.075).

**Table 2:** Main and interaction effects of bipolar disorder diagnosis and childhood maltreatment on the diagnoses of medical illnesses

	Adjusted OR^a^	95% confidence intervals		*p* value
		Lower	Upper	
At least one medical illness				
Main effects				
Bipolar disorder diagnosis	3.80	2.13	6.77	*<0.001*
Childhood maltreatment^b^	2.05	1.24	3.40	*0.006*
Child abuse^c^	2.14	1.23	3.70	*0.007*
Child neglect^d^	2.24	1.18	4.24	*0.013*
Interaction effects				
Bipolar disorder × childhood maltreatment	6.43	1.70	24.31	*0.006*
Bipolar disorder × abuse	6.31	1.46	27.24	*0.014*
Bipolar disorder × neglect	4.32	0.96	19.47	0.057
Number of medical illnesses				
Main effects				
Bipolar disorder diagnosis	4.81	2.71	8.53	*<0.001*
Childhood maltreatment	1.59	1.05	2.40	*0.028*
Child abuse	1.78	1.11	2.85	*0.016*
Child neglect	1.89	1.14	3.13	*0.014*
Interaction effects				
Bipolar disorder × childhood maltreatment	4.97	1.39	17.76	*0.014*
Bipolar disorder × abuse	5.23	1.32	20.75	*0.019*
Bipolar disorder × neglect	3.30	0.89	12.22	0.075

Significant *p* values are italicised

*OR* odds ratio derived from binary logistic regression for ‘at least one medical illness’ and from ordinal logistic regression for ‘number of medical illnesses’, *P* probability due to chance

^a^Adjusted for the effects of gender and age

^b^Childhood maltreatment was considered present if any type of child abuse or neglect were rated as moderate or severe

^c^Child abuse was considered present if any form of child abuse was rated as moderate or severe

^d^Child neglect was considered present if physical or emotional neglect was rated as moderate or severe

**Fig. 1:**
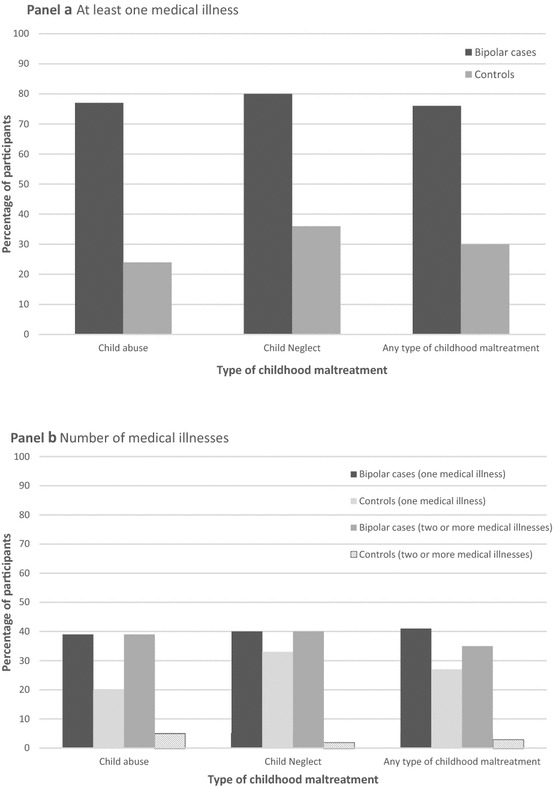
Percentage of participants with medical illnesses by type of childhood maltreatment experienced. The *y*-axis presents the percentage of participants with medical illnesses by the different types of childhood maltreatment groups (child abuse, child neglect and any type of childhood maltreatment) for bipolar cases and controls. **a** The diagnosis of at least one medical illness and **b** the number of medical illnesses (one and two or more)

Further examination of the interactions showed that exposure to any type of childhood maltreatment, child abuse and child neglect were significantly associated with higher odds of having at least one and a greater number of medical illnesses in the bipolar group but not for the controls (see Table 3). The percentage of bipolar cases and controls with medical illnesses by each type of childhood maltreatment is visually presented in Fig. 1. Given that there were a restricted number of participants that reported experiencing each type of child abuse (i.e. sexual, physical and emotional) and neglect (i.e. emotional and physical), analyses examining their individual and interactional effects were not possible.

**Table 3:** Relationship between childhood maltreatment and medical illnesses, presented separately for participants with bipolar disorder and controls

	Participants with bipolar disorder			Controls		
	Adjusted OR^a^	95% confidence interval	*p* value	Adjusted OR^a^	95% confidence interval	*p* value
At least one medical illness						
Any type of childhood maltreatment^b^	6.28	1.70–23.12	*0.006*	0.92	0.47–1.81	0.809
Child abuse^c^	4.99	1.32–18.75	*0.017*	0.81	0.36–1.84	0.616
Child neglect^d^	4.78	1.09–20.86	*0.038*	1.12	0.49–2.56	0.794
Number of medical illnesses						
Any type of childhood maltreatment	3.77	1.34–10.57	*0.012*	0.89	0.47–1.69	0.714
Child abuse	3.75	1.29–10.86	*0.015*	0.83	0.36–1.90	0.651
Child neglect	2.91	1.11–7.62	*0.029*	1.05	0.48–2.30	0.899

Significant *p* values are italicised

*OR* odds ratio derived from binary logistic regression for ‘at least one medical illness’ and from ordinal logistic regression for ‘number of medical illnesses’, *P* probability due to chance

^a^Adjusted for the effects of gender and age

^b^Childhood maltreatment was considered present if any type of child abuse or neglect were rated as moderate or severe

^c^Child abuse was considered present if any form of child abuse was rated as moderate or severe

^d^Child neglect was considered present if physical or emotional neglect was rated as moderate or severe

## Discussion

This study found a significant relationship between childhood maltreatment and medical illnesses in adulthood among individuals with bipolar disorder but not in unaffected controls. When the analyses were stratified by the type of childhood maltreatment the results were strongest for child abuse rather than child neglect. This is the first study to explore child neglect in this context using both controls and participants with bipolar disorder. Our findings are consistent with previous studies that have focused on mood disorders (Lu et al. 2008; McIntyre et al. 2012) and bipolar disorder specifically (Post et al. 2013). For instance, broadly defined childhood adversity, which includes child abuse (verbal, physical and sexual) and parental psychiatric diagnosis, but not child neglect, was found to be significantly associated with the overall number of medical illnesses in a sample of over 900 people with bipolar disorder (Post et al. 2013). Childhood adversity was also found to be significantly associated with specific medical illnesses in this group including arthritis, asthma, hyper- and hypo-tension (Post et al. 2013). However the results from the present investigation add to this literature by showing that the relationship between childhood maltreatment and medical illnesses is especially pertinent to bipolar disorder compared to controls.

Although the relationship between childhood maltreatment and medical illnesses in adulthood has been established in the general population (Scott et al. 2011), this relationship maybe particularly relevant to bipolar disorder for two reasons. First, high rates of childhood maltreatment have been found among people with bipolar disorder (Fisher and Hosang 2010; Palmier-Claus et al. 2016). Childhood maltreatment has also been associated with a worse clinical course among people with bipolar disorder, such as, earlier age of onset and more mood episodes (Agnew-Blais and Danese 2016). Such clinical course characteristics have also been linked to the medical morbidity in bipolar disorder (Magalhães et al. 2012). Bringing together these lines of research it is possible that childhood maltreatment may lead to an unfavourable clinical course in bipolar disorder that in turn contributes to the high medical burden observed in this illness. Although the exact mechanism that underpins this relationship is unclear, it has been postulated that it may reflect shared biological vulnerabilities, such as disruption in inflammation and oxidative systems (Magalhães et al. 2012). Alternatively, the more severe clinical course associated with childhood maltreatment (Agnew-Blais and Danese 2016) is likely to increase the need for medication treatment. The side effects of mood stabilisers and antipsychotics, include weight gain and insulin resistance (Newcomer 2007) are linked to various medical conditions, such as diabetes (Newcomer 2007), potentially explaining the link between childhood maltreatment and physical illnesses in bipolar disorder but may have also attenuated the results here. Although in the current study all of the participants with bipolar disorder were on long-term medication regimens for their psychiatric illness; therefore this confounding effect is unlikely here, but more research is needed to clarify this issue.

Second, the biological sequelae of childhood maltreatment, such as increased inflammation levels (Baumeister et al. 2016) are also evident in bipolar disorder (Leboyer et al. 2012) and various medical illnesses, particularly autoimmune diseases, such as arthritis and type I diabetes (Couzin-Frankel 2010). Research indicates that elevated inflammation is particularly pronounced among maltreated individuals with mood disorders even when compared to those who have a history of child abuse and neglect (Danese et al. 2008, 2011), thus potentially increasing the risk of such medical illnesses. For example, the results from one study found that inflammation levels were significantly higher among those with a history of childhood maltreatment and depression relative to those with depression only, childhood maltreatment only and controls (without either) (Danese et al. 2011). The biological consequences of childhood maltreatment in bipolar disorder warrants further research attention to better understand the possible mechanisms that underlie its high medical burden.

The results from the current investigation provide a novel contribution to the field by helping to show differential relationships between child abuse, child neglect and medical illnesses in bipolar disorder. Previous studies examined these adversities under one overarching construct of childhood maltreatment or did not explore the impact of child neglect separately (Lu et al. 2008; McIntyre et al. 2012; Post et al. 2013). The results of the present study suggest that the effect of child abuse on medical illnesses is not only significant but may also be stronger than that of child neglect in bipolar disorder. This is consistent with the results of previous studies which have shown that child abuse is associated with a list of medical disorders whereas neglect is linked to only a limited number of illnesses (Scott et al. 2011; Norman et al. 2012). It is possible that unhealthy lifestyles may explain the stronger association between the experience of child abuse and medical disorders compared to child neglect. For example, smoking is a major risk factor for a series of medical disorders (Ezzati et al. 2002), and has been significantly associated with child abuse but not child neglect (Norman et al. 2012). The exact mechanisms behind the link between child abuse and the medical burden in bipolar disorder is unclear and warrants further investigation. The limited sample size of the bipolar group may have impacted on the study’s power and is likely to have contributed to the non-significant interaction effect of child neglect and bipolar disorder status on the diagnosis of medical illnesses. Future research focusing on the biological, psychological and behavioural correlates of child abuse using larger samples would be especially informative.

With replication, the findings of this study are clinically valuable since they can be used to identify a subgroup of people with bipolar disorder (those with a history of childhood maltreatment) who are at risk of poor health (Post et al. 2013) and worse clinical course (Agnew-Blais and Danese 2016). These results underscore the need for routine assessment of childhood maltreatment history in clinical practice, which would assist with the early recognition of an ‘at risk’ group who would benefit most from targeted prevention and intervention efforts. Family therapy or psychoeducation focused on improving the social support provided to people with bipolar disorder maybe particularly beneficial. This suggestion is based on research that shows that social support influences the risk of relapse in bipolar disorder and mediates the effect of childhood maltreatment on physical health in adulthood (Herrenkohl et al. 2016).

There are a number of strengths of this study including the use of a well-characterised bipolar disorder sample and screened controls that completed validated instruments. But several limitations of the current study should be considered when interpreting the findings. First, the limited sample size of the bipolar group may reduce the power to detect significant effects and the ability to generalise our results. Participants with bipolar disorder in this study were recruited from across the UK through psychiatric outpatient clinics and self-help groups, so are not entirely biased or unrepresentative. Future studies should use a larger case–control sample to confirm the associations observed in this investigation. Second, childhood maltreatment and medical illnesses were assessed using self-report which has been associated with various problems (e.g. reporting accuracy) (Reuben et al. 2016). Retrospective self-report questionnaires used to assess childhood maltreatment are commonly used in both epidemiological and psychiatric studies (Norman et al. 2012; Agnew-Blais and Danese 2016). Moreover, childhood maltreatment data yielded from self-report show high concordance with case notes (Fisher et al. 2011) and therapists’ ratings (Bernstein et al. 2003). Substantial agreement between the self-report medical interview used here and the health practitioner reports of the diagnoses of medical disorders has been reported (Farmer et al. 2008). Nonetheless, it would be useful for future studies to replicate the findings of the present study using practitioner reports of medical illnesses, and prospective objective assessment of childhood maltreatment.

Finally, the incidence of several medical illnesses, particularly heart problems was relatively low especially compared to other studies, this precluded the examination of the specific association between childhood maltreatment and particular medical illnesses. This may have been the result of the age of the sample (median age 49 years, range 24–72 years), with the majority of participants outside of the median age of onset (58–64 years) for various heart problems, including coronary heart disease and stroke (Terry et al. 2004). But the prevalence of arthritis, hypertension and asthma in the current study is comparable to those reported in previous investigations (McIntyre et al. 2006; Perron et al. 2009). Future studies should explore the influence of childhood maltreatment on the medical morbidity in bipolar disorder using an older sample.

To summarise, this is one of a limited number of studies that has examined the relationship between childhood maltreatment and the medical morbidity in bipolar disorder. This study extends previous work by exploring the differential relationship between child abuse and neglect and in this context using a sample of controls and individuals with bipolar disorder. The results of this study showed that childhood maltreatment is significantly associated with medical ill health among people with bipolar disorder but not controls. On further examination of the data, child abuse showed the strongest association with medical illnesses compared to child neglect. With more research these findings can be used to identify individuals who would benefit most from prevention and intervention efforts.

## Abbreviations

SCAN: schedules for clinical assessment in neuropsychiatry interview
CTQ: Childhood Trauma Questionnaire
BADGE: gene-environment interplay in bipolar affective disorder
BaCCs: bipolar affective disorder case–control study

## References

[CR1] Agnew-Blais J, Danese A (2016). Childhood maltreatment and unfavourable clinical outcomes in bipolar disorder: a systematic review and meta-analysis. Lancet Psychiatry..

[CR2] Baumeister D, Akhtar R, Ciufolini S, Pariante CM, Mondelli V (2016). Childhood trauma and adult inflammation: a meta-analysis of peripheral C-reactive protein, interleukin-6 and tumour neurosis factor-α. Mol Psychiatry..

[CR3] Bernstein DP, Stein JA, Newcomb MD, Walker E, Pogge D, Ahluvalia T (2003). Development and validation of a brief screening version of the Childhood Trauma Questionnaire. Child Abuse Negl.

[CR4] Chang CK, Hayes RD, Perera G, Broadbent MTM, Fernandes AC, Lee WE (2011). Life expectancy at birth for people with serious mental illness and other major disorders from a secondary mental health care case register in London. PLoS ONE.

[CR5] Correll CU, Detraux J, De Lepeleire J, De Hert M (2015). Effects of antipsychotics, antidepressants and mood stabilizers on risk for physical diseases in people with schizophrenia, depression and bipolar disorder. World Psychiatry..

[CR6] Couzin-Frankel J (2010). Inflammation bares a dark side. Science.

[CR7] Crump C, Sundquist K, Winkleby MA, Sundquist J (2013). Comorbidities and mortality in bipolar disorder: a Swedish national cohort study. JAMA Psychiatry..

[CR8] Danese A, Caspi A, Williams B, Ambler A, Sugden K, Mika J (2011). Biological embedding of stress through inflammation processes in childhood. Mol Psychiatry..

[CR9] Danese A, Lewis S (2017). Psychoneuroimmunology of early-life stress: the hidden wounds of childhood trauma. Neuropsychopharmacology.

[CR10] Danese A, Moffitt TE, Pariante CM, Ambler A, Poulton R, Caspi A (2008). Elevated inflammation levels in depressed adults with a history of childhood maltreatment. Arch Gen Psychiatry.

[CR11] Ezzati M, Lopez AD, Rodgers A, Vander Hoorn S, Murray CJ (2002). Selected major risk factors and global and regional burden of disease. Lancet.

[CR12] Farmer A, Korszun A, Owen MJ, Craddock N, Jones L, Jones I (2008). Medical disorders in people with recurrent depression. Br J Psychiatry.

[CR13] Fisher H, Hosang G (2010). Childhood maltreatment and bipolar disorder: a critical review of the evidence. Mind Brain J Psychiatry..

[CR14] Fisher HL, Cohen-Woods S, Hosang GM, Korszun A, Owen M, Craddock N (2013). Interaction between specific forms of childhood maltreatment and the serotonin transporter gene (5-HTT) in recurrent depressive disorder. J Affect Disord.

[CR15] Fisher HL, Craig TK, Fearon P, Morgan K, Dazzan P, Lappin J (2011). Reliability and comparability of psychosis patients’ retrospective reports of childhood abuse. Schizophr Bull.

[CR16] Forty L, Ulanova A, Jones L, Jones I, Gordon-Smith K, Fraser C (2014). Comorbid medical illness in bipolar disorder. Br J Psychiatry.

[CR17] Gaysina D, Cohen-Woods S, Chow PC, Martucci L, Schosser A, Ball HA (2009). Association of the dystrobrevin binding protein 1 gene (DTNBP1) in a bipolar case–control study (BACCS). Am J Med Genet B Neuropsychiatr Genet..

[CR18] Gonzalez A (2013). The impact of childhood maltreatment on biological systems: implications for clinical interventions. Paediatr Child Health..

[CR19] Herrenkohl TI, Jung H, Klika JB, Mason WA, Brown EC, Leeb RT (2016). Mediating and moderating effects of social support in the study of child abuse and adult physical and mental health. Am J Orthopsychiatry.

[CR20] De Hert M, Correll CU, Bobes J, Cetkovich-Bakmas M, Cohen D, Asai I (2011). Physical illness in patients with severe mental disorders. I. Prevalence, impact of medications and disparities in health care. World Psychiatry..

[CR21] Hoang U, Stewart R, Goldacre MJ (2011). Mortality after hospital discharge for people with schizophrenia or bipolar disorder: retrospective study of linked English hospital episode statistics, 1999–2006. BMJ.

[CR22] Hosang GM, Fisher HL, Cohen-Woods S, McGuffin P, Farmer AE (2017). Stressful life events and catechol-*O*-methyl-transferase (COMT) gene in bipolar disorder. Depress Anxiety..

[CR23] Hosang GM, Johnson SL, Kiecolt-Glaser J, Di Gregorio MP, Lambert DR, Bechtel MA (2013). Gender specific association of child abuse and adult cardiovascular disease in a sample of patients with basal cell carcinoma. Child Abuse Negl.

[CR24] Hosang GM, Uher R, Maughan B, McGuffin P, Farmer AE (2012). The role of loss and danger events in symptom exacerbation in bipolar disorder. J Psychiatr Res.

[CR25] Kupfer DJ (2005). The increasing medical burden in bipolar disorder. JAMA.

[CR26] Leboyer M, Soreca I, Scott J, Frye M, Henry C, Tamouza R (2012). Can bipolar disorder be viewed as a multi-system inflammatory disease?. J Affect Disord.

[CR27] Lu W, Mueser KT, Rosenberg SD, Jankowski MK (2008). Correlates of adverse childhood experiences among adults with severe mood disorders. Psychiatr Serv.

[CR28] Magalhães PV, Kapczinski F, Nierenberg AA, Deckersbach T, Weisinger D, Dodd S (2012). Illness burden and medical comorbidity in the systematic treatment enhancement program for bipolar disorder. Acta Psychiatr Scand.

[CR29] McIntyre RS, Konarski JZ, Soczynska JK, Wilkins K, Panjwani G, Bouffard B (2006). Medical comorbidity in bipolar disorder: implications for functional outcomes and health service utilization. Psychiatr Serv.

[CR30] McIntyre RS, Soczynska JK, Liauw SS, Woldeyohannes HO, Brietzke E, Nathanson J (2012). The association between childhood adversity and components of metabolic syndrome in adults with mood disorders: results from the international mood disorders collaborative project. Int J Psychiatry Med.

[CR31] Newcomer JW (2007). Antipsychotic medications: metabolic and cardiovascular risk. J Clin Psychiatry.

[CR32] Norman RE, Byambaa M, De R, Butchart A, Scott J, Vos T (2012). The long-term health consequences of child physical abuse, emotional abuse, and neglect: a systematic review and meta-analysis. PLoS Med..

[CR33] Palmier-Claus JE, Berry K, Bucci S, Mansell W, Varese F (2016). Relationship between childhood adversity and bipolar affective disorder: systematic review and meta-analysis. Br J Psychiatry.

[CR34] Perron BE, Howard MO, Nienhuis JK, Bauer MS, Woodward AT, Kilbourne AM (2009). Prevalence and burden of general medical conditions among adults with bipolar I disorder: results from the national epidemiologic survey on alcohol and related conditions. J Clin Psychiatry.

[CR35] Post RM, Altshuler LL, Leverich GS, Frye MA, Suppes T, McElroy SL (2013). Role of childhood adversity in the development of medical co-morbidities associated with bipolar disorder. J Affect Disord.

[CR36] Reuben A, Moffitt TE, Caspi A, Belsky DW, Harrington H, Schroeder F (2016). Lest we forget: comparing retrospective and prospective assessments of adverse childhood experiences in the prediction of adult health. J Child Psychol Psychiatry Allied Discipl.

[CR37] Scott D, Happell B (2011). The high prevalence of poor physical health and unhealthy lifestyle behaviours in individuals with severe mental illness. Issues Ment Health Nurs..

[CR38] Scott KM, Von Korff M, Angermeyer MC, Benjet C, Bruffaerts R, de Girolamo G (2011). Association of childhood adversities and early-onset mental disorders with adult-onset chronic physical conditions. Arch Gen Psychiatry.

[CR39] Shannon C, Hanna D, Tumelty L, Waldron D, Maguire C, Mowlds W (2016). Reliability of reports of childhood trauma in bipolar disorder: a test–retest study over 18 months. J. Trauma Dissociation..

[CR40] Slopen N, Kubzansky LD, McLaughlin KA, Koenen KC (2013). Childhood adversity and inflammatory processes in youth: a prospective study. Psychoneuroendocrinology..

[CR41] Terry DF, Wilcox MA, McCormick MA, Perls TT (2004). Cardiovascular disease delay in centenarian offspring. J Gerontol Ser A..

[CR42] Wing JK, Babor T, Brugha T, Burke J, Cooper JE, Giel R (1990). Schedules for clinical assessment in neuropsychiatry. Arch Gen Psychiatry.

